# Antilisterial Effectiveness of *Origanum vulgare* var. *hirtum* and *Coridothymus capitatus* Essential Oils and Hydrolates Alone and in Combination

**DOI:** 10.3390/foods13060860

**Published:** 2024-03-12

**Authors:** Serena D’Amato, Chiara Rossi, Francesca Maggio, Luca Valbonetti, Vincenzo Savini, Antonello Paparella, Annalisa Serio

**Affiliations:** 1 Asl Pescara, Veterinary Hygiene Service for Food of Animal Origin, Via Paolini 47, 65124 Pescara, Italy; serena-damato@libero.it; 2 Department of Bioscience and Technology for Food, Agriculture and Environment, University of Teramo, Via R. Balzarini 1, 64100 Teramo, Italy; crossi@unite.it (C.R.); fmaggio@unite.it (F.M.); lvalbonetti@unite.it (L.V.); aserio@unite.it (A.S.); 3 Asl Pescara, Clinical Microbiology and Virology Unit, Spirito Santo Hospital, Via Fonte Romana 8, 65124 Pescara, Italy; vincenzo.savini@asl.pe.it

**Keywords:** hydrolate, essential oil, *Listeria monocytogenes*, checkerboard, interaction, CLSM

## Abstract

The antimicrobial activity of *Origanum vulgare* var. *hirtum* (O) and *Coridothymus capitatus* (C) essential oils (EOs) and hydrolates (HYs) of the same botanical species was evaluated on sixteen *L. monocytogenes* strains from food and clinical origins. The antimicrobial activity was assessed by Minimum Inhibitory Concentration (MIC) determination, viable cell enumeration over time up to 60 min, and evaluation of the cellular damage through Confocal Laser Scanning Microscope (CLSM) analysis. EOs exhibited antimicrobial activity with MIC values ranging from 0.3125 to 10 µL/mL. In contrast, HYs demonstrated antimicrobial effectiveness at higher concentrations (125–500 µL/mL). The effect of HYs was rapid after the contact with the cells, and the cell count reduction over 60 min of HY treatment was about 1.2–1.7 Log CFU/mL. *L. monocytogenes* cells were stressed by HY treatment, and red cell aggregates were revealed through CLSM observation. Moreover, the combinations of EOs and HYs had an additive antilisterial effect in most cases and allowed the concentration of use to be reduced, while maintaining or improving the antimicrobial effectiveness. The combined use of EOs and HYs can offer novel opportunities for applications, thereby enhancing the antimicrobial effectiveness and diminishing the concentration of use. This provides the added benefit of reducing toxicity and mitigating any undesirable sensory effects.

## 1. Introduction

Since ancient times, aromatic plants, also known as herbs and spices, have been used as food flavorings, but also because of their preservative and medicinal properties [1]. *Coridothymus capitatus* and *Origanum vulgare* subsp. *hirtum*, both of which are extensively acknowledged aromatic herbs, belong to the taxonomic categorization of the Lamiaceae family [2]. Across various cultures and traditions, aromatic herbs have been largely utilized as a delightful and nutritionally superior alternative to the conventional use of salt (NaCl) in culinary practices, thereby fostering healthier dietary habits and promoting overall well-being. In addition to their culinary applications, these herbs have also been widely acknowledged for their potential therapeutic properties, thus serving as valuable constituents in the formulation of health supplements, further augmenting their significance in the realm of human health.

*C. capitatus* and *O. vulgare* are rich in phytonutrients, minerals, and vitamins essential for maintaining good health. These compounds are recognized for their disease-preventive and health-enhancing characteristics, which are recognized as a valuable part of the herbs’ benefits [3]. For instance, incorporating *O. vulgare* subsp. *hirtum* into the diet could potentially offer a significant source of quercetin [2]. Conversely, *C. capitatus* contains vitamins A, C, B-complex, K, and E [3].

Recently, plant derivatives such as EOs, and their co-products HYs, have received considerable attention due to their antimicrobial and antibiofilm activities [4]. EOs and HYs are composed of different bioactive compounds, known as the phytocomplex. The antimicrobial effectiveness of the phytocomplex is the result of the interaction among these molecules, as a consequence of the type and abundance of the chemical compounds, which act on cellular targets [4]. Besides their antimicrobial activity, they possess advantages over antibiotics by hindering antimicrobial resistance in bacteria; in fact, it is stated that it is more difficult for bacteria to develop resistance to multicomponent EOs than to antibiotics, which are often composed of single molecular entities [5]. By definition, EOs are complex mixtures of organic, volatile, and lipophilic compounds [6], with an intense sensory “fingerprint”. Recently, EOs have been studied as natural preservatives and sanitizers in foods and food environments [7], and as natural antimicrobials and antifungals in clinical applications [8,9]. Nevertheless, EOs require special warnings when used per os or topically, owing to the high toxicity of many terpene compounds [10]. EOs may be toxic and pose a cancer risk due to their high macro- and microelement contents [11]. Additionally, EOs can contain harmful persistent organic pollutants and pesticides that are detrimental to human health [12]. Furthermore, when applied in food systems at high concentrations, EOs can negatively affect the sensory characteristics of food products, causing unpleasant tastes [6]. To overcome these limits, the amounts of EOs added to foods should be reduced [13].

A potential strategy could involve the replacement of EOs or their combination with hydrolates. HYs, also called aromatic waters, hydrosols, or floral waters, are the co-products of EO distillation, and can be described as hydrophilic solutions that contain less than 1 g/L of water-soluble aromatic compounds from EOs [14]. The volatile compounds of HYs are mainly monoterpene alcohols, sesquiterpene alcohols, aldehydes, and ketones [15], depending on the EOs’ origin. Interestingly, a recent study argued that at equal concentration, the HY volatiles are relatively more effective as microbial inhibitors than those of EOs [10]. As HYs do not have strong sensory traits and are normally less toxic than EOs, they may provide a good alternative to EOs for both food and clinical applications. In addition, being considered by-products of the aromatic plant manufactory, HYs can represent a sustainable strategy, according to the principles of the circular economy, to face the environmental issues derived from waste production [16]. However, the physical-chemical properties and biological characteristics of HYs have the potential to suggest more extensive application of these compounds, although the quantities employed may need to be increased to effectively attain the desired objectives.

Given this context, this study aimed to reduce the EOs concentrations of use necessary for antimicrobial activity. It evaluated the antimicrobial effectiveness of EOs and HYs from *Origanum vulgare* var. *hirtum* and *Coridothymus capitatus*, both individually and in combination, against strains of *Listeria monocytogenes* sourced from food and clinical origins.

Strains of different sources were investigated to obtain a more complete framework of the effectiveness of EOs and HYs.

## 2. Materials and Methods

### 2.1. Antimicrobial Compounds

Commercial and food-grade *C. capitatus* and *O. vulgare* var. *hirtum* EOs (CEO, OEO) and HYs (CHY, OHY) were kindly provided by Exentiae S.r.l. Soc. Agricola (Catania, Italy). The EOs and HYs were stored at 4 °C in dark colored glass bottles until the analysis. According to Rossi et al. [7], starting from the pure commercial oils, the EO emulsions were prepared at the initial concentration of 40.0 μL/mL through dilution in Phosphate Buffer Saline (PBS) 10 mmol/L, pH 7.4, added to 10 μL/mL of Tween 80 (Sigma-Aldrich, Milan, Italy). On the contrary, the HYs were employed without any dilution. To determine the antibiotic profile, eight antimicrobial agents were tested: ampicillin, ciprofloxacin, chloramphenicol, gentamicin, penicillin, rifampicin, trimethoprim, and vancomycin (Sigma-Aldrich, Milan, Italy), at different concentrations.

### 2.2. Bacterial Strains and Culture Collection

Sixteen strains of *L. monocytogenes* (Table 1) were selected from the collection of the Faculty of Bioscience and Technology for Food, Agriculture and Environment of the University of Teramo (Italy) and of Santo Spirito Hospital, Pescara (Italy). The strain set consisted of type strain ATCC 19114 (n = 1) and strains from food (n = 8) and clinical (n = 7) origins. The strains were stored at −80 °C in Brain Heart Infusion (BHI; Oxoid Thermo Fisher Scientific, Rodano, Italy) and glycerol (20.0% *v*/*v*; Sigma Aldrich, Milan, Italy), added as a cryoprotectant. Before each experiment, the bacterial strains were cultivated overnight at 37 °C in BHI Agar (Oxoid Thermo Fisher Scientific, Rodano, Italy). The inocula were prepared by taking a colony from the Petri dish and resuspending it in 1 mL of BHI broth, incubating at 37 °C for 18 h to reach the early stationary phase. After the incubation period, the cells were harvested by centrifugation, washed three times with PBS 10 mmol/L, pH 7.4, and finally resuspended in 1 mL of the same buffer. The inocula were standardized at OD_620nm_ (0.08–0.1), then diluted with sterile BHI to reach about 10^6^ CFU/mL, and the loads were confirmed by means of plate counts on Agar Listeria according to Ottaviani Agosti (ALOA, Oxoid Thermo Fisher Scientific, Rodano, Italy) incubated at 37 °C for 48 h.

### 2.3. Minimum Inhibitory Concentration Determination

Based on the CLSI protocol [19], the antimicrobial activity of the EOs and HYs was tested by MIC determination in a 96-well microtitre plate (Corning incorporated, Kennebunk, ME, USA) using the broth microdilution technique. For the EOs, the concentrations tested ranged from 20 to 0.078 µL/mL; for HYs, the concentration range was 500–1.95 µL/mL. For the antibiotics, the concentrations used ranged from 512 to 0.13 µg/mL. For the analyses, the strains were suspended in Mueller–Hinton Broth (Oxoid Thermo Fisher Scientific). The MIC value was considered as the lowest concentration of EOs, HYs, and antibiotics at which no microbial growth was observed after incubation at 37 °C for 48 h. Positive controls (inoculum in BHI) and negative controls (BHI without inoculum and EOs and HYs) were included in each experiment. The experiments were replicated three times.

### 2.4. Time-Kill Kinetics Assay

The Time-Kill Kinetics assay (TKK) assesses the in vitro activity of an antimicrobial agent against a bacterial strain over time [20]. TKK was performed according to the CLSI guidelines [21] on three *L. monocytogenes* strains (ATCC 19114, LM6, and L3), chosen as representative on the basis of the MIC results. In detail, *L. monocytogenes* ATCC 19114 was chosen as a reference strain, while LM6 was chosen for its sensitivity to HYs among food-origin strains. On the other hand, L3 was chosen for its resistance to HYs among clinic-origin strains. Five hundred microliters of HYs were used to treat 500 µL of the *L. monocytogenes* population at 10^6^ CFU/mL in BHI broth. The HYs were tested at the MIC values, which were 125, 250, and 500 µL/mL for ATCC 19114, LM6, and L3, respectively. An untreated *L. monocytogenes* inoculum for each strain was used as positive control. All the samples were incubated at 37 °C for 60 min, and the living populations were quantified after definite time intervals (0, 5, 15, 30, 45, and 60 min). In detail, aliquots (100 µL) removed from each tube were centrifuged at 13,000 rpm for 5 min (Eppendorf centrifuge 5415D; Hauppauge, NY, USA), washed three times with PBS 10 mmol/L, pH 7.4, serially diluted, and then plated into ALOA plates. After incubation at 37 °C for 48 h, the number of colony forming units (CFU/mL) was determined. The experiment was performed in triplicate.

### 2.5. Confocal Laser Scanning Microscope Analysis

CLSM was used to investigate membrane integrity and morphological changes of *L. monocytogenes* strains treated with HYs. The cells were stained using the LIVE/DEAD BacLight Bacterial Viability Kit (Molecular Probes, OR; Thermo Fisher Scientific, Waltham, MA, USA), according to the suggested protocol of the manufacturer [22]. For this experiment, the same *L. monocytogenes* strains used in the TKK assay (the previous section) were analyzed. Briefly, 3 μL of a mixture of SYTO 9 and propidium iodide (PI) (1:1) was added to the *L. monocytogenes* cells (10^6^ CFU/mL), previously treated or not with HYs at the MICs (125 µL/mL for ATCC 19114, 250 µL/mL for LM6, and 500 µL/mL for L3) for different times (0, 5, 15, 30, 45, and 60 min). The samples were incubated for 15 min, in the dark at room temperature. Then, all the samples were centrifuged at 13,000 rpm for 5 min and washed three times with physiological solution (0.85% NaCl) to remove the dyes excess. Fifteen microliters of the stained *L. monocytogenes* strains were applied on glass slides, sealed with coverslips, and observed through CLSM.

*L. monocytogenes* cells were observed using a Nikon A1R confocal imaging system (Nikon Corp., Tokyo, Japan) and controlled by the Nikon NIS Elements interface, equipped with a Plan Apo λ 100× Oil objective (Numerical Aperture: 1.4; Refractive Index: 1.515). The excitation/emission values for the dyes were 488/525–50 nm and 561.5/595–50 nm for SYTO9 and PI [7], respectively. Triplicate scans were undertaken on each slide.

### 2.6. Synergistic Interaction Evaluation

In the combination assay, the checkerboard method described by Fratini et al. [23] was applied with some modifications to evaluate the synergistic action of the HYs with the EOs. Five *L. monocytogenes* strains (ATCC 19114, L315, LM6, LM13, and L3), selected according to the MIC results, were used. Both HYs were matched with EOs of the same and different botanical species. Serial microdilutions of the antimicrobial agents were prepared following the same procedure used to evaluate the MICs, and HYs/EOs were dispensed into 96-well microtiter plates in a checkerboard manner. Each well was inoculated with 10^6^ CFU/mL of *L. monocytogenes* and incubated at 37 °C for 48 h. The combinations were analyzed by calculating the Fractional Inhibitory Concentration Index (FICI), which enables an understanding of whether the relationship between the two active agents used simultaneously on a microorganism is synergistic, additive, indifferent, or antagonistic [10]. The FICI was obtained using Equation (1):

(1) $${FIC}_{A}={MIC}_{A}\;in\;combination/{MIC}_{A}\;alone,\;{FIC}_{B}={MIC}_{B}\;in\;combination/{MIC}_{B}\;alone,\;FICI={FIC}_{A}+{FIC}_{B}$$

The FICI was used to interpret the effects of the interactions between HYs and EOs as a synergistic effect (FICI ≤ 0.5), additive effect (0.5 < FICI ≤ 1), indifferent effect (1 < FICI ≤ 4), or antagonistic effect (FICI > 4) [24].

### 2.7. Data Analysis

MIC and FICI results were superimposable in the different replicates; thus, only TKK results were subjected to ANOVA (XLSTAT ver. 2017), by pairwise comparison between the control and the treated cells at the same incubation time, employing Tukey’s test (* *p* < 0.05).

## 3. Results and Discussion

### 3.1. EOs and HYs Chemical Composition

The chemical composition of EOs and HYs is shown in Table 2. Carvacrol was the primary constituent of CEO and CHY, at 73.0% and 100%, respectively, while for OEO and OHY, thymol was revealed as the main bioactive compound, accounting for 44.17% and 100%, respectively. In OEO, other important compounds were γ-terpinene (26.09%) and p-cymene (16.03%), which are both precursors of thymol and present biological activities [25,26].

### 3.2. Minimum Inhibitory Concentration Determination

MIC values for antibiotics (µg/mL), and EOs and HYs (µL/mL), were determined after 48 h of incubation at 37 °C by broth microdilution assay. In Table S1, the antimicrobial activity of the antibiotic compounds is shown in terms of MICs, which range from 512 to 0.031 µg/mL. The response to antibiotic treatment varied among the *L. monocytogenes* strains, indicating a strain-specific susceptibility. Notably, the clinical strains displayed a lower level of resistance, except for L3, resulting in a multi-resistant strain, with MIC values ranging from 2 to 256 µg/mL, and being resistant to ampicillin, penicillin, rifampicin, vancomycin, and trimethoprim. Conversely, the food strains exhibited a higher level of resistance, particularly to ampicillin, penicillin, and vancomycin, with MICs ranging from 0.031 to >512 µg/mL. Instead, as shown in Table 3, the EOs exhibited higher antibacterial effectiveness than their co-products against the sixteen tested *L. monocytogenes* strains. The MIC values for EOs ranged from 0.3125 to 10 µL/mL, and L317, L291, and L239 were the most sensitive strains (MIC 0.3125–0.625 µL/mL). In general, the MIC results revealed the higher resistance of food strains, compared to clinical strains, to the EOs’ antimicrobial action. The MICs of the food strains were between 0.625 and 10.0 µL/mL, whereas the range of clinical strains was 0.312–2.5 µL/mL, with the exception of L368 (MIC range 5.0–10.0 µL/mL). The lower sensitivity of food strains to different EOs has been already demonstrated, probably because aromatic plants and spices, as well as antioxidants and flavorings, containing the main constituents of *O. hirtum* and *C. capitatus* EOs, are commonly used in the human diet and during food processing. As a consequence, this exposure could reduce the sensitivity to EOs in the strains living in food environments. *L. monocytogenes* may face several kinds of environmental stresses in the food chain, which serve as pre-exposure adaptation [27]. These stresses include physical stressors such as heat, high pressure, desiccation, and irradiation; chemical stressors, such as acids, salts, and oxidants; and biological stressors, such as microbial antagonism, which induces the bacterial cross-protection response that generates cells with increased resistance to the same or other types of stresses [28]. Cross-protection, an extensively observed phenomenon in microorganisms, refers to the adaptive response of cells to a mild or sublethal condition, followed by their subsequent exposure to a lethal stress. This particular process, in turn, serves to considerably augment the tolerance level of cells when confronted with a variety of stress conditions [29]. However, as a result of their multifaceted antimicrobial capabilities and their inability to promote bacterial resistances, EOs continue to hold great potential for the application in the food supply chain.

The HYs’ MIC values ranged from 125 to 500 µL/mL, with the lowest MIC value (125 µL/mL) for ATCC 19114 and L315. Unexpectedly, at the tested concentrations, CHY was ineffective against the LM4 strain. Although the EOs exhibited the broadest spectrum activity against *L. monocytogenes*, the results obtained for the HYs are noticeable, especially considering their aqueous nature and the fact that they are usually applied without any dilution. CHY was more effective in counteracting the development of food strains, while OHY was more efficient in controlling strains of clinical origin. Currently, HYs are in the spotlight as an antimicrobial alternative. In fact, in spite of the relatively high concentration required to exert antimicrobial activity (i.e., 125–500 µL/mL), the amount of their bioactive compounds is generally low, and the sensory profile is mild [7].

For the subsequent analyses, ATCC 19114 was selected as the reference strain, while LM6 and L3 were chosen among the food and clinical strains for their good resistance to HYs.

### 3.3. Time-Kill Kinetics Assay

**Figure 1:**
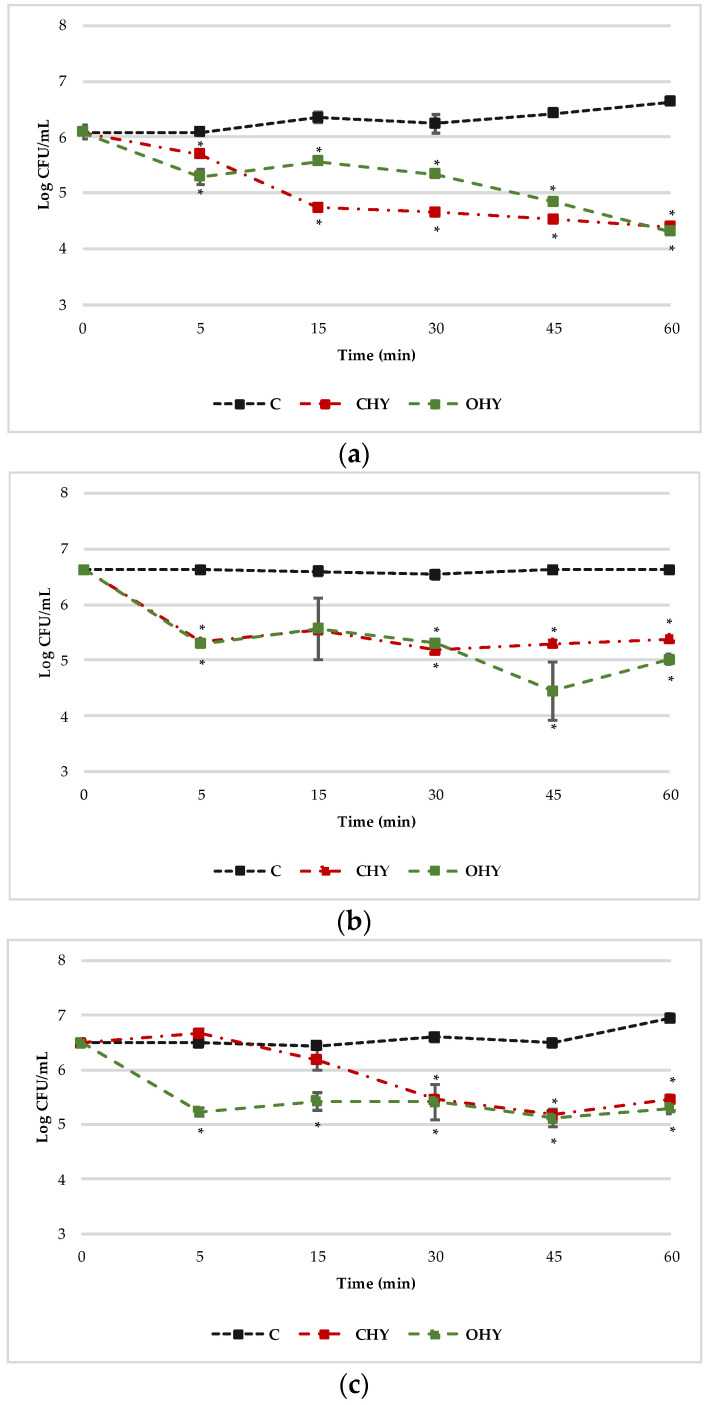
TKK curves of CHY and OHY at MICs (125 µL/mL for ATCC 19114, 250 µL/mL for LM6, and 500 µL/mL for L3) against *L. monocytogenes* ATCC 19114 (**a**), LM6 (**b**), and L3 (**c**). Results are expressed as the average of three replicates and the bars represent the standard deviations. The asterisks indicate a statistically significant difference (* *p* < 0.05) between control and the treatments for each analysis time. CHY, *C. capitatus* hydrolate; OHY, *O. hirtum* hydrolate.

After confirming the inhibitory activity of HYs against the tested strains, the TKK was investigated. Figure 1 summarizes the TKK results of the HYs at the MICs against *L. monocytogenes* strains ATCC 19114, LM6, and L3. In general, TKK showed that HYs significantly decreased the viable cell numbers of *L. monocytogenes* in a time-dependent and strain-dependent manner. Compared with the ATCC 19114 control group, CHY and OHY showed an inhibitory effect, with a 0.4 and 0.8 Log reduction, respectively, after only 5 min of exposure, about 1.6 and 0.9 Log after 15–30 min, 1.9 and 1.6 Log after 45 min, and a similar Log reduction (2.24 and 2.32) at the end of the experimental time. For the LM6 strain, the two HYs showed similar load values during the treatment, with Log reductions ranging from 1.0 to 1.6, except after 45 min of exposure, where the Log reductions for CHY and OHY were 1.3 and 2.2, respectively. The decrease in viable cell numbers of L3 exerted by the HYs was different depending on the botanical species. In fact, for OHY, a significant decrease in viable counts of *L. monocytogenes* was observed starting from 5 min of treatment (1.3 Log reduction); on the contrary, CHY reached similar reductions after 45 min of treatment. To summarize, in 60 min of exposure, HYs reduced the cellular load of each *L. monocytogenes* strain tested by about 1.2–1.7 Log CFU/mL. The antimicrobial effectiveness of HYs could be related to the presence of the bioactive monoterpenes, carvacrol and thymol. Given their lipophilicity, terpenes have great potential to traverse cell walls, and the presence of a hydroxyl group and a delocalized electron system cause the destabilization of the bacterial membrane integrity [30,31]. The significant reduction in the number of viable cells in a short time (60 min), starting immediately after cells’ exposure to HYs, indicates that HYs are interesting solutions for surface disinfection, both in food and clinical environments. EOs have already been demonstrated to be effective immediately after the contact with the cells, whereas similar data were missing for HYs. In fact, the application of natural compounds to decontaminate surfaces that come into contact with food has been extensively investigated by numerous researchers [31,32]. These authors have proposed EOs as an emerging technology for the sanitization of food-contact surfaces, due to their remarkable ability to prevent and regulate the growth of planktonic and sessile cells of several food-borne pathogens such as *L. monocytogenes*, *E. coli*, and *S. aureus*. Our results add new data suggesting that HYs should also be considered among these strategies.

To acquire further information about the effect of HYs against *L. monocytogenes*, the cell membrane integrity was also examined.

### 3.4. Confocal Laser Scanning Microscope Observation

**Figure 2:**
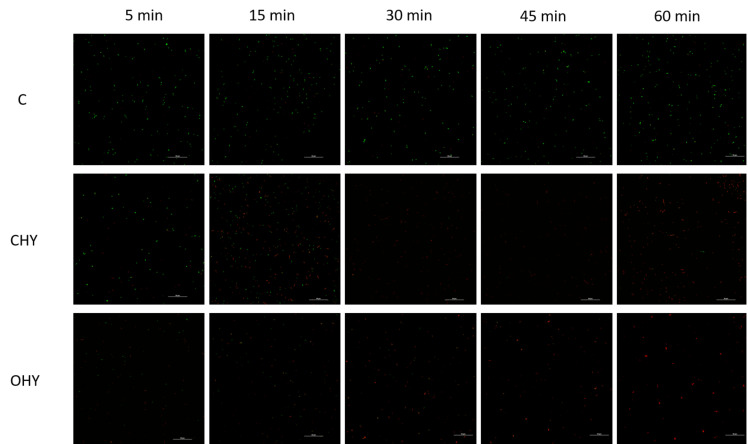
Representative CLSM images of untreated *L. monocytogenes* L3 cells and cells exposed to CHY and OHY (500 µL/mL) over 60 min. C, control; CHY, *C. capitatus* hydrolate; OHY, *O. hirtum* hydrolate.

Bacterial cell viability and membrane damage were observed by CLSM combined with SYTO9 (green) and propidium iodide (PI, red) probes. For the untreated *L. monocytogenes* L3 cells, selected as the representative strain, no major damage was shown (Figure 2). On the contrary, the exposure of the strain to HYs at the MIC value (500 µL/mL) resulted in significant membrane damage that increased with exposure time. In fact, after 5 min of treatment, a small number of red fluorescent cells were detected, suggesting that the cell membrane was damaged. After 15 min, a marked increase in red-colored cells was observed, and, in the following exposure times, few or no green fluorescent cells were discovered. Furthermore, the presence of red cells in aggregates was observed for OHY-treated cells, particularly after 60 min of exposure. The staining with the SYTO9 and PI probes confirmed that the CHY and OHY treatments affected cell viability and membrane integrity. CLSM images showed a diffuse red discoloration of the treated cells over 60 min, suggesting damaged cytoplasmic membranes or cellular death. This information is important, as it clarifies that the treatment applied is bactericidal and not bacteriostatic. The diffuse aggregation of the *L. monocytogenes* cells after the OHY contact could be a response to the stress encountered [33,34]. In fact, cells that are stressed or damaged, but still alive, can aggregate to reduce the exposed surface. Thus, the most external cells are more exposed, therefore preserving the inner ones [33,34]. Moreover, EOs are hydrophobic and can thus also increase cell hydrophobicity, favoring the adhesion of each cell with the neighbor. A further explanation was provided by Hollander and Yaron [35], who demonstrated how EOs and their hydrophobic constituents damage the cytoplasmic membrane, causing protein leakage that induces cell aggregation [36].

### 3.5. Evaluation of the Synergistic Interaction of EO and HY Pairs

The combined effect of HYs and EOs on the antimicrobial efficacy against *L. monocytogenes* was evaluated by the checkerboard method, and the results are reported in Table 4. The tested strains were selected according to the MIC results; in particular, ATCC 19114 was selected as the reference strain, L315 for its HY sensitivity, LM6 for its moderate HY sensitivity, and LM13 and L3 for their good resistance. The combination of the two EOs and their HYs mainly produced an additive antimicrobial effect against the *L. monocytogenes* strains tested (in 18 strains out 20), with FICI values between 0.500 and 1000. An indifferent effect was detected only by the application of the CEO/CHY pair against *L. monocytogenes* ATCC 19114. Although synergistic interactions were observed only in one case (with OEO/CHY pair against *L. monocytogenes* L315), the low FICI values observed are relevant, since the simultaneous use of two substances, with different characteristics, allows them to be used at lower percentages than those used for the single EO and HY applications. Few similar data are available in the literature, but the results deserve further investigation. HYs and EOs together showed a relevant effectiveness, probably due to the combination of the active molecules contained in the respective formulations. Interestingly, the only synergistic effect was observed for the combination of HY and OE of the different botanical species. In this case, the combination of the antimicrobic compounds could have boosted the antimicrobial effect, confirming what has already been observed in numerous studies [13,37]. Combining active substances with synergistic interactions aims to decrease the amount of each compound and enhance the biological activity against a specific target [38]. Moreover, although this result was only observed for one strain, and is therefore strain-dependent, it has to be highlighted that L315 was among the most sensitive strains to HYs alone, but not to EOs alone.

## 4. Conclusions

In conclusion, our results demonstrate that the combination of EOs and HYs can open up new perspectives for application, boosting the antimicrobial effect. In fact, in view of a practical use of these natural substances as fast antimicrobials for controlling pathogenic bacteria in both food and clinical environments, the possibility of using low doses of EOs and HYs may reduce any undesired sensory effects and toxicity. In fact, strong odor, insolubility in aqueous foods, and sensitivity to heat and environmental conditions during food processing and storage are disadvantages that are driving the reduction in EO concentrations in food formulations. Moreover, before developing applications in the food industry, any possible toxicological issue should be carefully investigated to ensure that the new formulations can be considered safe in any conditions of use of the finished product.

Alternatively, in clinical environments, the application of EOs in surface disinfection is hampered by their hydrophobicity, while their combination with hydrolates would make them easier to apply and rinse. Subsequent research endeavors will yield additional insights into the impacts of combining EOs and HYs within food and clinical settings.

## Figures and Tables

**Table 1:** Source of the *L. monocytogenes* strains investigated in this study.

Strain	Origin	References
ATCC 19114	Type strain	
LM1	Cured pork meat	Unpublished
LM2	Deli meat sandwich	Unpublished
LM4	Deli meat sandwich	[17]
LM6	Pork ribs	[6]
LM12	Cured pork meat	Unpublished
LM13	Pork meat	Unpublished
LM17	Salami	[17]
LM19	Pork meat	[17]
L3	Clinical environments	[18]
L253		
L239		
L291		
L315		
L317		
L368		

**Table 2:** Details on *C. capitatus* and *O. vulgare* subsp. *hirtum* EOs and HYs.

Botanical Species	Chemical Component	EO [%]	HY ^1^ [%]	Origin	Cultivation Method
*Coridothymus capitatus*	Carvacrol	73.0	100	South coast of Sicily	Organic Farming ^2^
	p-Cymene	9.48			
	β-Caryophyllene	5.1			
	γ-Terpinene	4.30			
	Terpinolene	1.70			
	β-Thujene	1.61			
	β-Myrcene	1.37			
	α-Pinene	1.20			
	β-Linalool	0.47			
	Borneol	0.4			
	α-Phellandrene	0.33			
	L-Terpinen-4-ol	0.3			
	Camphene	0.26			
	Thymol	0.20			
	β-Pinene	0.16			
	p-Mentha-1,3,8-triene	0.03			
*Origanum vulgare* subsp. *hirtum*	Thymol	44.17	100		
	γ-Terpinene	26.09			
	p-Cymene	16.03			
	Terpinolene	3.66			
	Isothymol methyl ether	2.96			
	β-Thujene	1.81			
	β-Myrcene	1.36			
	α-Pinene	0.95			
	β-Caryophyllene	0.73			
	O-Methylthymol	0.69			
	Terpinen-4-ol	0.44			
	α-Phellandrene	0.41			
	δ-Cadinene	0.18			
	β-Pinene	0.13			
	β-trans-Ocimene	0.12			
	Aromadendrene	0.10			
	γ-Muurolene	0.09			
	Camphene	0.08			

^1^ HYs contained 0.7% EO. ^2^ Guidelines for Good Agricultural and Wild Collection Practice (GACP) of Medicinal and Aromatic Plants by EUROPAM (European Herb Growers Association).

**Table 3:** Minimum Inhibitory Concentrations (MIC, µL/mL) of *C. capitatus* and *O. hirtum* EOs and HYs against sixteen *L. monocytogenes* strains after 48 h of incubation at 37 °C.

Strain	EOs		HYs	
	CEO	OEO	CHY	OHY
ATCC 19114	1.25	2.5	125	125
LM1	10.0	10.0	125	500
LM2	10.0	10.0	125	500
LM4	5.0	10.0	n.e. ^1^	250
LM6	2.5	2.5	250	250
LM12	10.0	10.0	125	500
LM13	2.5	2.5	500	500
LM17	1.25	0.625	250	125
LM19	0.625	1.25	250	250
L315	1.25	2.5	125	125
L253	2.5	0.625	250	250
L317	0.625	0.3125	250	125
L291	0.625	0.625	500	250
L239	0.3125	0.3125	250	125
L368	5.0	10.0	250	125
L3	1.25	1.25	500	500

^1^ n.e: not effective at the tested concentration. Each value is the mean of three replicates. EOs, essential oils; HYs, hydrolates; CEO, *C. capitatus* essential oil; OEO, *O. hirtum* essential oil; CHY, *C. capitatus* hydrolate; OHY, *O. hirtum* hydrolate.

**Table 4:** Fractional Inhibitory Concentration Index (FICI) of the EO–HY pairs against *L. monocytogenes* strains and their type of interaction.

Combination EO/HY	Strain	FIC		FICI	Outcome
		EO	HY		
CEO/CHY	ATCC 19114	0.500	1.000	1.500	Indifferent
	LM6	0.500	0.250	0.750	Additive
	LM13	0.500	0.250	0.750	Additive
	L315	0.500	0.500	1.000	Additive
	L3	0.500	0.500	1.000	Additive
OEO/OHY	ATCC 19114	0.500	0.250	0.750	Additive
	LM6	0.500	0.500	1.000	Additive
	LM13	0.500	0.250	0.750	Additive
	L315	0.500	0.062	0.562	Additive
	L3	0.500	0.500	1.000	Additive
CEO/OHY	ATCC 19114	0.250	0.500	0.750	Additive
	LM6	0.500	0.250	0.750	Additive
	LM13	0.500	0.125	0.625	Additive
	L315	0.500	0.500	1.000	Additive
	L3	0.500	0.500	1.000	Additive
OEO/CHY	ATCC 19114	0.500	0.500	1.000	Additive
	LM6	0.500	0.500	1.000	Additive
	LM13	0.500	0.250	0.750	Additive
	L315	0.500	0.007	0.507	Synergistic
	L3	0.500	0.250	0.750	Additive

Type of interaction: synergistic if FICI ≤ 0.5, additive if 0.5 < FICI ≤ 1, indifferent if 1 < FICI ≤ 4, and antagonistic if FICI > 4.

## Data Availability

The original contributions presented in the study are included in the article/Supplementary Materials, further inquiries can be directed to the corresponding author.

## Supplementary Materials

The following supporting information can be downloaded at: https://www.mdpi.com/article/10.3390/foods13060860/s1. Table S1: Overview of the antibiotic profile shown by the *L. monocytogenes* strains. Table S2: Gas-chromatographic characterization of *Coridothymus capitatus* and *Origanum vulgare* subsp. *hirtum* essential oils (EO) and hydrolates (HY).
