# Supplementary material for: Antilisterial Effectiveness of Origanum vulgare var. hirtum and Coridothymus capitatus Essential Oils and Hydrolates Alone and in Combination
Source: Foods. 2024 Mar 12;13(6):860. doi: 10.3390/foods13060860 (PMC10969000; doi:10.3390/foods13060860)
Supplement: Supplementary file 1 [file foods-13-00860-s001.zip › foods-2887616-supplementary.pdf]

Table S1. Overview of the antibiotic profile shown by the *L. monocytogenes* strains.

STRAIN	Ampicillin	Ciprofloxacin	Chloramphenicol	Gentamicin	Penicillin	Rifampicin	Trimethoprim	Vancomycin
ATCC19114	256	1	16	8	512	16	8	64
LM1	8	2	32	32	0.25	0.25	4	0.5
LM2	2	1	32	4	0.125	1	4	1
LM4	1	1	8	16	8	1	0.25	0.25
LM6	>512	1	32	32	512	16	8	64
LM12	2	1	64	64	2	2	4	1
LM13	16	16	32	2	32	0.031	2	16
LM17	>512	1	16	32	512	16	8	64
LM19	512	1	32	16	512	8	8	64
**L315**	<1	2	16	8	<1	<0.13	<0.13	2
L253	<1	2	16	16	<1	<0.13	<0.13	2
L317	<1	2	16	8	<1	<0.13	<0.13	2
L291	<1	1	16	64	1	<0.13	<0.13	2
L239	2	2	16	64	8	16	4	8
L368	<1	2	16	16	<1	0.13	0.13	1
L3	256	2	32	16	512	16	32	32

The results were expressed in μg/mL. The standard deviation is not reported, being zero for all replicates.

Table S2. Gas-chromatographic characterization of *Coridothymus capitatus* and *Origanum vulgare* subsp. *hirtum* essential oils (EO) and hydrolates (HY).

(RT= Retention Time; Area= area of the peak; Result (%)= percentage composition).

	Coridothymus capitatus							***Origanum vulgare* subsp. *hirtum***					
	**EO**			**HY**				**EO**			**HY**		
**COMPOUNDS**	**RT**	**AREA**	**Result [%]**	**RT**	**AREA**	**Result [%]**	**COMPOUNDS**	**RT**	**AREA**	**Result [%]**	**RT**	**AREA**	**Result [%]**
β-Thujene	6.522	1.01E+09	1.61				β-Thujene	6.511	9.21E+08	1.81			
α-Pinene	6.77	7.46E+08	1.20				α-Pinene	6.760	4.83E+08	0.95			
Camphene	7.34	1.62E+08	0.26				Camphene	7.332	4.08E+07	0.08			
β-Pinene	8.328	9.67E+07	0.16				β-Pinene	8,319	6.81E+07	0.13			
β-Myrcene	8.733	8.54E+08	1.37				β-Myrcene	8.728	6.94E+08	1.36			
α-Phellandrene	9.434	2.05E+08	0.33				α-Phellandrene	9.428	2.10E+09	0.41			
Terpinolene	9.83	1.06E+09	1.70				Terpinolene	9.818	1.86E+09	3.66			
p-Cymene	10.163	5.91E+09	9.48				p-Cymene	10.154	8.17E+09	16.03			
p-Mentha-1,3,8-triene	10.328	1.68E+07	0.03				β-trans-Ocimene	10.676	6.15E+07	0.12			
γ-Terpinene	11.549	2.70E+09	4.32				γ-Terpinene	11.543	1.33E+10	26.09			
β-Linalool	13.465	2.94E+08	0.47				Terpinen-4-ol	17.111	2.23E+08	0.44			
Borneol	16.645	2.65E+08	0.42				O-Methylthymol	19.259	3.52E+08	0.69			
L-Terpinen-4-ol	17.09	2.12E+08	0.34				Isothymol methyl ether	19.643	1.52E+09	2.96			

Thymol	22.175	1.36E+08	0.22			Thymol	22.142	2.25E+10	44.17	22.165	100
Carvacrol	22.551	4.56E+10	73.04	22.463	100	β-Caryophyllene	27.319	3.70E+08	0.73		
β-Caryophyllene	27.319	3.15E+09	5.05			γ-Muurolene	29.677	4.37E+07	0.09		
						Aromadendrene	31.244	5.06E+07	0.10		
						δ-Cadinene	31.434	9.65E+07	0.18		
**TOTAL**			**100**		**100**	**SUM**			**100**		**100**
